# Genetic Manipulation for Improved Nutritional Quality in Rice

**DOI:** 10.3389/fgene.2020.00776

**Published:** 2020-07-24

**Authors:** Priyanka Das, Sanghamitra Adak, Arun Lahiri Majumder

**Affiliations:** Division of Plant Biology, Bose Institute, Kolkata, India

**Keywords:** nutrition, rice, biotechnology, breeding, micronutrient

## Abstract

Food with higher nutritional value is always desired for human health. Rice is the prime staple food in more than thirty developing countries, providing at least 20% of dietary protein, 3% of dietary fat and other essential nutrients. Several factors influence the nutrient content of rice which includes agricultural practices, post-harvest processing, cultivar type as well as manipulations followed by selection through breeding and genetic means. In addition to mutation breeding, genetic engineering approach also contributed significantly for the generation of nutrition added varieties of rice in the last decade or so. In the present review, we summarize the research update on improving the nutritional characteristics of rice by using genetic engineering and mutation breeding approach. We also compare the conventional breeding techniques of rice with modern molecular breeding techniques toward the generation of nutritionally improved rice variety as compared to other cereals in areas of micronutrients and availability of essential nutrients such as folate and iron. In addition to biofortification, our focus will be on the efforts to generate low phytate in seeds, increase in essential fatty acids or addition of vitamins (as in golden rice) all leading to the achievements in rice nutrition science. The superiority of biotechnology over conventional breeding being already established, it is essential to ascertain that there are no serious negative agronomic consequences for consumers with any difference in grain size or color or texture, when a nutritionally improved variety of rice is generated through genetic engineering technology.

## Introduction

Nutrition is the foremost requirement for an organism to operate vital functions such as growth, development, and reproduction. Plants acquire nutrition through the process of photosynthesis using H_2_O and CO_2_, in the presence of light. Apart from H and C, 15 other elements are important for the development of major plant species (White and Brown, 2010). Furthermore, macronutrients (S, P, Mg, Ca, K, N, O, C, H) and micronutrients (Mo, Ni, Cu, Zn, Mn, B, Fe, Cl) are also essential parts of plants nutrition (White and Brown, 2010). Being incapable of photosynthesis, animals including human beings depend upon plants for supporting their nutrition requirement either directly or indirectly.

Plant nutrients fulfilling human needs are called phytonutrients and different plants may contain different nutrients of altered levels. Hence, a single plant is not enough to gratify human nutritional needs. In recent times, contemporary agriculture is focusing on human nutritional improvement by generating improved varieties of staple crops such as rice, wheat, maize and grain legumes (Ricachenevsky et al., 2019). Grain legumes fulfill the requirement of protein and cereal crops have a high level of carbohydrate which offers ∼80% of calorie intake (Lafiandra et al., 2014; Shewry and Hey, 2015). Yet, cereals or legumes lack sources of important micro- and macro-nutrients, which can result in serious human health problems. Hence, a major part of ongoing staple plant research is focusing on the enhancement of the nutritional value of crops with aiming to increase the micro- and macro-nutrients. Rice is the major staple food crop for about half of the world’s population whose nutritional status is a topic of discussion in the research sector. This principal staple food crop contains a reduced quantity of many essential micro- and macro- elements such as vitamins, minerals, some phytochemicals, essential amino acids and fatty acids, which are indispensable to human health (Birla et al., 2017). Elevation of nutritional quality of rice has been a great effort over the last decades either by traditional breeding, marker assisted breeding or by genetic or transgenic methods.

Human nutrition is completely dependent on plant systems directly or indirectly. Efforts have been taken for long to improve the nutritional quality of staple crops as these are the main sources of food with a low amount of micronutrients. The application of genomics to nutrition science is called “nutritional genomics” or “nutrigenomics” where identification of plant genes with nutritional importance is a significant step (DellaPenna, 1999). Precisely the discipline nutrigenomics refers to the interplay between plant biochemistry, genomics and nutrition in humans (DellaPenna, 1999). In the present review, we intend to focus on the importance of rice crop in terms of its nutritional value, health benefits, and need for enhancement of rice nutrition through biotechnology and conventional and/or molecular breeding approach.

## Nutritional Value and Health Benefits of Rice

Rice is the chief source of carbohydrate and is used as an integral part of a balanced diet for billions of people in the world. Rice is the extensively grown food crop occupying about millions hectares of farmlands (Muthayya et al., 2014). Rice grain has a high biological value for its high energy and high calories. Beyond the classification of long grain and short-grain rice types, other known types of rice are brown rice, white rice, polished rice, organic rice, wild rice, puffed rice, colored rice, etc. Brown and white rice are preferentially used as food by the major parts of the world population.

A comparison of nutrients of different staple foods is provided in Table 1. Apart from carbohydrates, rice contains a very low amount of phosphorous, iron and protein. It also has some quantity of calcium. In comparison to other food crops like maize, wheat, tuber roots and legumes, rice grain is also very low in mineral content (Mahender et al., 2016) and a small amount of vitamin B. Most of the nutrients and minerals are concentrated in the outer brown layer of the grain. Rice bran from the brown layer is an important source of nutrients such as vitamins, proteins, minerals and antioxidants (tocotrienols and gamma oryzanol) (Tian et al., 2004; Iqbal et al., 2005; Schramm et al., 2007). Hence, brown rice is the most nutritious among all types of rice. Comparative value of the nutrient composition between brown rice and white rice has been given in Table 2.

**TABLE 1:** Comparison of nutrients in different staple foods (per 100 g) (FAO http://www.fao.org/3/t0567e/T0567E0d.htm).

Nutrients	Brown rice	Wheat	Maize	Potato
Protein (g)	7.3	10.6	9.8	2
Fat (g)	2.2	1.9	4.9	0.1
Carbohydrate (g)	71.1	61.6	60.9	15.4
Carotene (mg)	0	0.02	0.37	0
Thiamine (mg)	0.29	0.45	0.32	0.11
Riboflavin (mg)	0.04	0.1	0.1	0.05
Vitamin E (mg)	0.8	1.4	1.9	0.06
Iron (mg%)	3	4	3	0.08
Zinc (mg)	2	3	3	0.3
Lysine (g/16 g N)	3.8	2.3	2.5	6.3
Threonine (g/16 g N)	3.6	2.8	3.2	4.1
Methionine + cystine (g/16 g N)	3.9	3.6	3.9	3.6
Tryptophan (g/16 g N)	1.1	1	0.6	1.7

**TABLE 2:** Composition of nutrients in rice (per a cup of white or brown rice).

Product	Brown rice	White rice
Protein (g)	5.54	4.43
Fat (g)	1.96	0.39
Carbohydrate (g)	51.7	53.2
Fiber (g)	3.23	0.56
Calories (Kcal)	248	242
Iron (mg)	1.1	2.8
Phosphorus (mg)	208	68.8
Magnesium (mg)	78.8	24.2
Zinc (mg)	1.4	0.8
Copper (mg)	0.2	0.1
Manganese (mg)	2.0	0.7
Thiamine	0.4	0.3
Vitamin B-6 (mg)	0.3	0.1
Niacin	5.2	3.4
Folate (mcg)	18.2	108

*Newsletter, Medically reviewed by Natalie Butler, R.D., L.D. (2019) – Written by Megan Ware, RDN, LD.*

Rice supplies complex carbohydrates that are broken down into glucose and the major part of glucose is used as energy and as vital fuel for the brain. Brown rice has a slow starch digestibility and some starch is never turned into sugar at all and reaches the large intestine intact (Dolson, 2009). Hence, Type II diabetic rice eaters would be better off eating brown rice instead of white rice. Eating brown or whole grain rice is known to lower down the risk of diabetes (Sun et al., 2010).

Rice is an exceptional food for a balanced diet as it has no cholesterol, fat or, sodium. Its’ bran contains up to 80% fatty acids. Rice oils comprise the unsaturated fatty acid such as linoleic acid and oleic acid which cannot be synthesized by humans but are essential in maintaining the cell membranes and the nervous system functions (Frei and Becker, 2004). Rice is a significant source of protein having eight of the necessary amino acids in a balanced amount which play a great role in healthy hair and skin, better eyesight, heart and lungs nourishment, better nervous system and brain function. Recently, a comparison of rice bran protein was carried out with vegetable and animal protein. The digestibility index explains that rice bran protein was significantly higher (94.8%) than rice endosperm protein. Other sources like soy protein (91.7%) and whey protein (92.8%) exhibited comparatively lower digestibility than rice bran protein (Han et al., 2015).

The B-complex vitamins (riboflavin, niacin and thiamine) of brown rice support nourishment to blood vessels and skin. Rice bran also contains beneficial anti-oxidants of the Vitamin E family (Lloyd et al., 2000). Rice tocopherols and tocotrienols are found to have anti-cancer activities (Lloyd et al., 2000; Kline et al., 2004). Rice oryzanols have the capacity to lessen the absorption of cholesterol (Lloyd et al., 2000). It has also been investigated that tocotrienol of rice bran can stop blood clots which may lead to strokes (Frei and Becker, 2004). Furthermore, red rice bran is loaded in tannins and anthocyanins which have anti-inflammatory properties. In addition, tannins have anti-bacterial effects and known to prevent cardiovascular diseases and cancer (Chung et al., 1998; Redondo et al., 2014). Black and red rice are rich in iron and zinc (Ahuja et al., 2008) which are needed for hemoglobin production and enzymatic processes respectively. Rice also provides important minerals like potassium, manganese and copper which are needed in the human body for normal metabolism, brain and nerve functions. Minerals in natural brown rice have a role in nourishing the hormonal system, regulating blood pressure and heal wounds. Rice also provides iron, phosphorus and potassium along with other nutrients to improve the bloodstream and to uphold inner water balance and internal harmony.

## Need of Improvement of the Nutritional Quality of Rice

Micronutrients are required for a healthy human being and their recommended daily allowances are presented in Table 3. Micronutrient deficiency is a major problem for the rice eaters. Frequently, rice eaters increase the intake of rice without increasing other nutritionally enriched foods when there is a calorie demand. Many people do not opt for a nutritious diet that includes whole grains, protein, vegetables and fruits.

**TABLE 3:** Micronutrients required for healthy human population (adapted from FAO/WHO, 2001, Human Vitamin and Mineral Requirements).

Recommended nutrient intake for human health			
Micronutrient types	Infants (0–6 months)	Adults	Pregnancy
Thiamin (mg/day)	0.2	1.2	1.4
Riboflavin (mg/day)	0.3	1.3	1.4
Niacin (NEs/day^*a*^)	2	16	18
Vit B6 (mg/day)	0.1	1.3	1.9
Folate (μg/day-RNI*)	80	400	600
Vit B12 (μg/day-RNI*)	0.4	2.4	2.6
Vit C (mg/day-RNI*)	25	45	55
Vit A (μg RE/day^+^)	375	500	800
Vit D (μg/day-RNI*)	5	10	5
Calcium (mg/day)	300	1000	1200
Iodine (μg/kg/day)	15	2	3.5
Iron (mg/day)	0.55	0.6	100
Zinc (mg/day)	0.84	3.37	5

*NEs/day^a^ niacin equivalents. RNI^∗^ Reccomended nutrient intake. RE^+^ Estimated mean requirement.*

**FIGURE 1:**
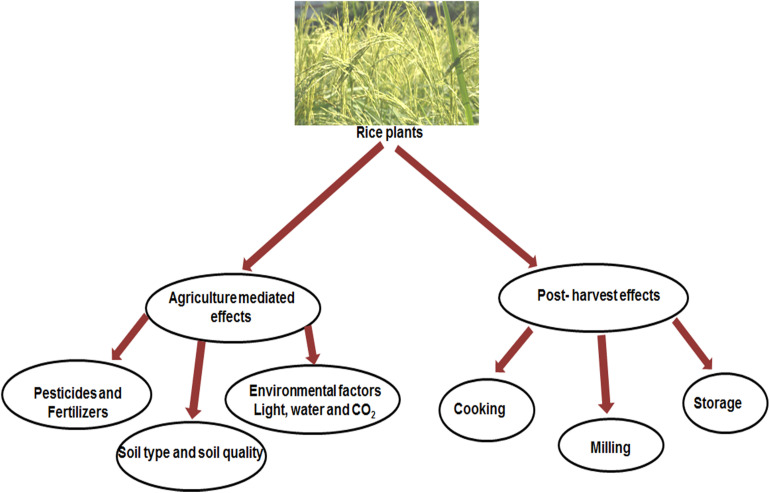
Nutrient composition of rice as affected by various practices.

Outer wall to inner endosperm of rice contains different level of nutrients (Juliano, 1993). Although rice is a good source of vitamin B group, riboflavin, thiamine and niacin and aspartic- and glutamic acids, rice contains a low level of lysine (Grist, 1986; Juliano, 1993). Recent climate change has turned out a decrease in the nutrient content of rice as well as other cereal crops (Smith and Myers, 2019). A Rising level of CO_2_ in the atmosphere decreased the levels of protein, micronutrients and B vitamins in rice which may impact a negative effect on fetal and child health in the rice dependant countries. The rice grown in increased CO_2_ concentrations contained 17% decreased vitamin B1, 17% decreased vitamin B2, 13% decreased vitamin B5 and 30% decreased vitamin B9 than the rice grown under regular CO_2_ concentrations. Average decline of 5% in zinc, 8% in iron and 10% in protein have been reported (Smith and Myers, 2019). A number of factors influence the nutrient composition of rice as outlined in Figure 1. Research at International Rice Research Institute (IRRI) shows that nutrient content and composition can differ considerably between different varieties (Juliano and Villareal, 1993; Graham et al., 1999). Experiments under controlled environment showed that solar rays, soil nitrogen, extent of plant maturation and fertilizer application can alter protein, iron and zinc content (Graham et al., 1999; Iwata, 2002). Nitrogen level and soil type have effect on iron and zinc content in rice grains (Senadhira et al., 1998).

**FIGURE 2:**
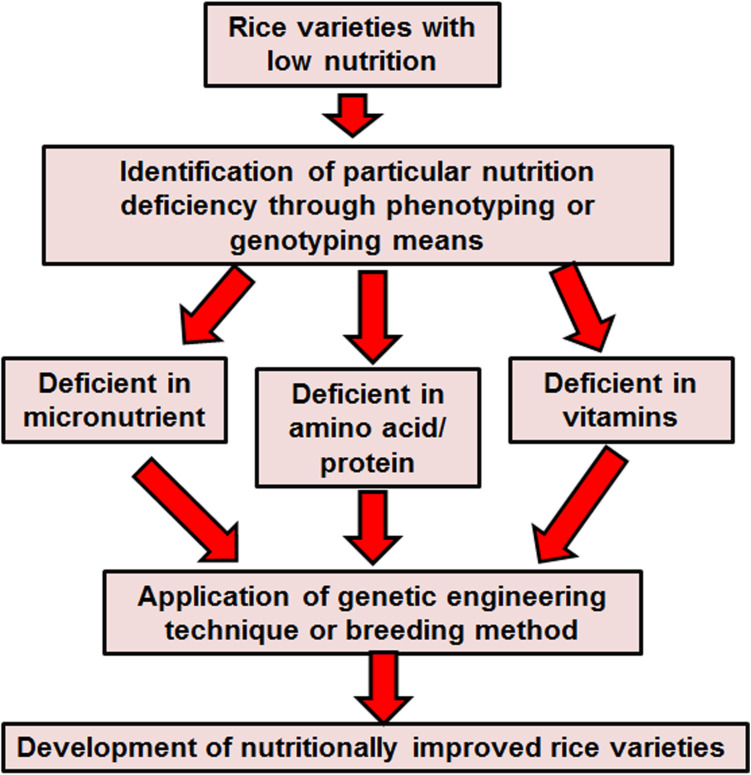
Strategies for enhancement of nutrients in rice.

After harvesting the rice, storage, processing, washing and cooking practices can all influence its nutritional value, however, post-harvest losses are hardly ever taken into consideration for nutritional evaluation. The indirect effect of post-harvest nutrient losses in rice can have a deep impact on food safety. Post-harvest loss is a quantifiable qualitative and quantitative loss in a specified product (Grewal and Sangha, 1990; Malik and Chaudhary, 2002). After harvesting, paddy rice is dehydrated and is then milled to eliminate the uneatable hull. Hulled rice or brown rice is composed of 2–3% embryo, 90% endosperm and 6–7% bran (Chen et al., 1998). Further elimination of the bran gives white rice. Paddy rice produces 65% white rice, 10% bran and 25% hulls (Saunders and Betschart, 1979). In short, with further elimination of the bran layer more minerals and vitamins are lost (Malik and Chaudhary, 2002). Cleansing of rice, before cooking, leads to lose 2–7% protein, 11–26% riboflavin, 22–59% thiamine, 20–60% niacin and 20–41% potassium (Juliano, 1993). The cooking process of rice in India leads to a loss of 50% calcium and phosphorus, 10% protein and 75% iron. Cooking with excess water tends to lose more than 25% of riboflavin, thiamine and niacin (Saunders and Betschart, 1979). Frying rice with high temperatures can annihilate more than 50% of thiamine (Saunders and Betschart, 1979). Increasing the content of iron, zinc and provitamin A carotenoid in rice endosperm can lessen these nutrient deficiencies, particularly among the rural and urban poor people who do not have access to enriched foods and diversified diets. Hence, it is necessary to improve the nutritional quality of rice through the traditional breeding or emerging biotechnology method. Plant biologists adopted several approaches to meet this goal. A schematic strategy has been presented in Figure 2.

Yet, it is the topic of discussion that whether only an increase in micronutrient or amino acid or protein in the rice endosperm can considerably fulfill the nutrition requirement in rice dependant people. The nutritional status of the crop may also depend on proper growth of the plant and heavily influenced by the environmental conditions. Abiotic stresses such as salinity, drought and temperature extremes are other limiting factors for rice growth, development and yield. Hence, to adopt the micronutrient- or essential amino acid-rich rice varieties, the superior traits are preferred to be combined with other traits such as abiotic stress tolerance.

## Manipulation at Amino Acid and Protein Level

Humans have a great interest in increasing the levels of some essential amino acids in crop plants because of their incapability to produce the same. Crop genetic studies and genetic engineering technology wonderfully helped to succeed in enriching the important crop plants through enhancement of their essential amino acid and protein content. Specifically, lysine and tryptophan got much attention as their production is limiting in cereal crops. Genetic breeding methods have ended in comparatively limited success in some crop species due to a shortage of adequate quality genetic resources for plant breeding. Furthermore, genetic traits for high levels of lysine and tryptophan are generally linked with abnormal growth of the plant as these characters do not function in a seed-specific manner. On the other hand manipulation through genetic engineering approach worked great as it could allow seed-specific expression of specific characters of interest. An additional benefit of genetic engineering method is that the trait can be introduced into multiple plant species and genotypes. Yet, the advancement of metabolic pathways through genetic engineering too needs a thorough understanding of the interaction of regulatory network pathways that fine-tune plant growth and yield.

## Manipulation of Lysine Content

**FIGURE 3:**
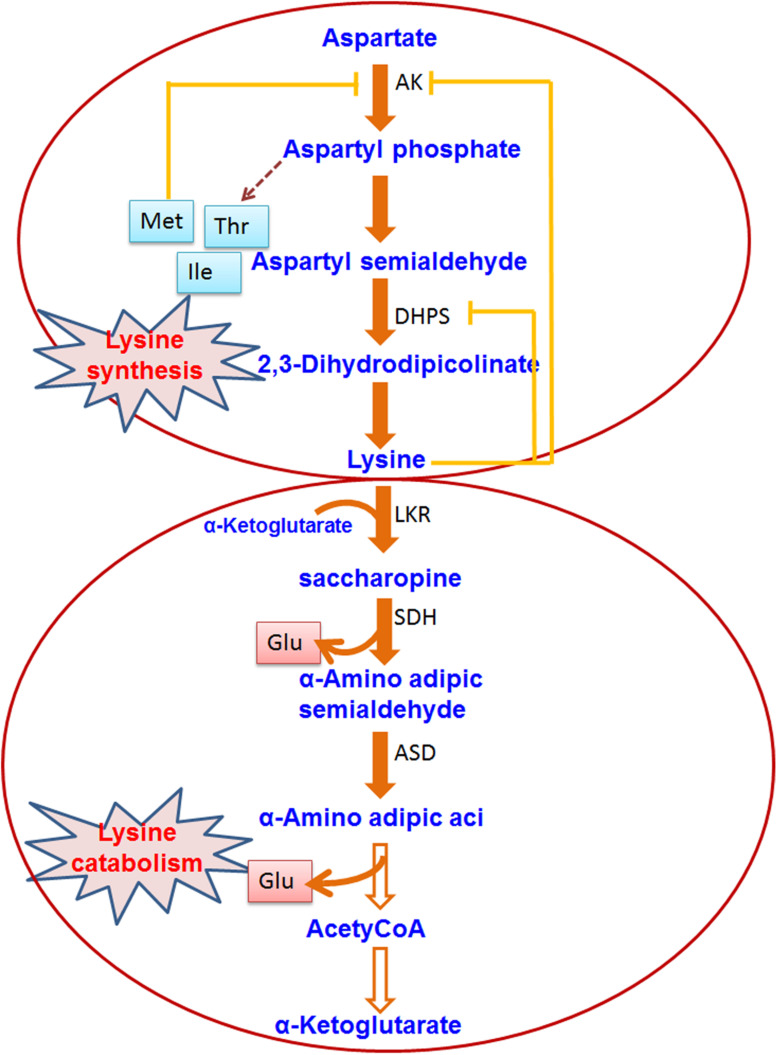
Lysine biosynthesis and catabolism in plants. The pathway shows the biosynthesis/catabolism of Lysine. Enzymes involved in the pathway are indicated by abbreviations. The hollow/dashed arrow indicates non-specified enzymatic reactions (AK, Aspartate kinase; DHPS, dihydrodipicolinate synthase; LKR, lysine ketoglutaric acid reductase; SDH, saccharopine dehydropine dehydrogenase; ASD, aminoadipic-semialdehyde dehydrogenase).

Lysine is an important amino acid for plants but its production in rice and other major crop plants is very low. Therefore, many of the rice researchers were devoted to understand the regulation of lysine metabolism and its utilization to increase the level of free lysine in the seeds. Some researchers are also interested in using the proteins which have high lysine content. Lysine is produced through a branch of the aspartate family pathway which also leads to the synthesis of two other essential amino acids, i.e., methionine and threonine (Galili, 2002; Figure 3). Fluctuation during the lysine biosynthesis part is regulated by a feedback inhibition ring where the activity of dihydrodipicolinate synthase (DHDPS) is inhibited by lysine, the earliest enzyme exclusively dedicated to the biosynthesis of lysine (Galili, 2002). Genetic mutations in the tobacco *DHDPS* or constitutive expression of a bacterial lysine-insensitive DHDPS in transgenic tobacco or Arabidopsis plants caused overproduction of lysine in all plant parts (Negrutiu et al., 1984; Frankard et al., 1992; Shaul and Galili, 1992). High levels of lysine in all plant tissues can result in abnormal vegetative and reproductive growth, and reduced seed yield (Frankard et al., 1992; Shaul and Galili, 1992). For increasing cereal lysine content, an additional technique has been adapted in recent times which includes the introduction of lysine at alternative codons at the time of translation by using a recombinant tRNA-lysine (Wu and Chen, 2003). Recombinant expression of this tRNA in transgenic rice could enhance seed lysine content significantly (Wu and Chen, 2003). Stable expression of Arabidopsis Lys tRNA synthetase gene in maize plants caused significant enrichment of lysine content in the grain (Wu et al., 2007). Yang et al. (2016) targeted bacterial aspartate kinase and dihydrodipicolinate synthase into the intragenic region in the rice genome. Two pyramid transgenic lines (designated as High Free Lysine; HFL1 and HFL2) were obtained with ∼25-fold higher lysine content in the seeds than the WT plants. The transgenic lines showed improved physicochemical properties with unchanged starch composition. Field performance showed a slight alteration in the height of the plant and seed color. Mature rice seeds with engineered HFL possessed the dark-brown appearance of endosperm. Yang et al. (2018) adopted transcriptomic and metabolomic approach and found increased serotonin biosynthesis which is linked with the color of endosperm in HFL rice. It was further confirmed by the overexpression of a key gene of serotonin biosynthesis, i.e., TDC3. It was evident from the study of Yang et al. (2018) that TDC expression and jasmonate signaling pathway were sturdily induced in the late phase of endosperm growth in HFL rice, corresponding with serotonin build up and dark-brown color development. This study is promoting the efforts to generate a nutritionally appreciative crop. In a different approach, Liu et al. (2016) took a lysine-rich protein (LRP) gene from *Psophocarpus tetragonolobus* using an endosperm-specific promoter. The transgenic rice seeds revealed to have 30% more lysine than the WT plants. In addition, transgenic seeds have an increased level of other amino acids in comparison to the WT plant. The hybrid of the transgenic rice lines also revealed increased lysine content in the seeds. Two endogenous lysine-rich histone proteins (RLRH1 and RLRH2) of rice were over-expressed in rice seeds to achieve lysine biofortification (Wong et al., 2015). The level of lysine in the seed of transgenic rice plants was increased up to 35%, and other essential amino acids stayed balanced, as required by the dietary standards of the World Health Organization. Pseudo-cereal *Amaranthus hypochondriacus* has seed albumin (AmA1) rich in all the essential amino acids. The introduction of recombinant AmA1 in rice revealed increased (1.06∼12.87%) level total protein contents in the seeds of the transgenic plants than the wild type (Xu et al., 2017). Additionally, the level of other essential amino acids (e.g., lysine, valine, and threonine) was also increased significantly in the transgenic lines in comparison to the wild type counterpart (Xu et al., 2017). Binding protein is a molecular chaperon protein which works together with unfolded secretory proteins in the endoplasmic reticulum domain and supports their folding. Kawakatsu et al. (2010) showed that over-accumulation of lysine-rich binding protein (BiP) in transgenic rice plants resulted in high lysine germplasm and it offered a special strategy to increase the lysine content of the cereal grain.

## Manipulation of Threonine Content

In addition to low lysine, rice grains have also a very low level of threonine. Increase the threonine content has been shown to be intimately related to the lysine content in terms of regulation and several attempts have been made to achieve such feat. In a synthetic biology approach (Jiang et al., 2016) two genes were artificially synthesized by combining endogenous rice genes with lysine (K)/threonine (T) motif (TKTKK) coding stretches. They were named as *TKTKK1* and *TKTKK2* with their respective proteins having 73.1 and 83.5% of lysine and threonine. Independent introduction of these two genes into the rice genome resulted in rice seeds with a significant increase in lysine (33.87%), threonine (21.21%), total amino acids (19.43%) and crude protein (20.45%) content as compared to wild type control plants (Jiang et al., 2016).

## Manipulation of Tryptophan Content

Another limiting essential amino acid in cereals is tryptophan. The synthesis of tryptophan in plants is feedback regulated at the anthranilate synthase step responsible for its biosynthesis. Kreps et al. (1996) studied that insensitivity of anthranilate synthase to feedback inhibition by tryptophan enhances tryptophan accumulation. This study showed the way to use this trait to increase the level of tryptophan in cereals. Expression of analogous feedback-insensitive *a*-subunit of the rice anthranilate synthase caused a significant increase in the level of free tryptophan in the seeds but it also had negative effects on vital agronomical traits such as spikelet fertility, yield and germination (Wakasa et al., 2006). The feedback-insensitive *a*-subunit of the rice anthranilate synthase also revealed increased level the free tryptophan content in transgenic potato, *Solanum tuberosum* (Yamada et al., 2004; Ishihara et al., 2006), adzuki bean *Vigna angularis* (Hanafy et al., 2006) and Arabidopsis. A mutation-breeding approach has been used to produce tryptophan-accumulating rice by gene targeting mediated mutagenesis of OASA2-an *a*-subunit of anthranilate synthase (Saika et al., 2011). Transgenic rice plants were generated successfully harboring OASA2 with S126F/L530D, Y367A/L530D, and Y367A mutations. The generated plants possessed 230- fold higher level of tryptophan without any apparent morphological or developmental changes in the plant. Mutant gene Mtr1 (mtr1-D) encodes a rice prephenate dehydratase with a point mutation in the protein regulatory section. Yamada et al. (2008) showed that this mutant gene encoding an altered phenylalanine biosynthetic enzyme results in the accumulation of phenylalanine and tryptophan when introduced in rice callus.

## Manipulation of Cysteine and Methionine Content

The Sulfur amino acid composition has a decisive effect on seed protein quality. Methionine, homocysteine, cysteine and taurine are the four common sulfur-containing amino acids, but only methionine and cysteine are incorporated into proteins. Sulfur-rich protein from sunflower seed albumin (SSA) was identified and introduced in rice to alter cysteine and methionine contents in the grain. Despite the increase in total protein content in the transgenic plants, an analysis of 7% of total seed protein of transgenic rice grains showed very little change in the amount of total sulfur or organic sulfur as compared to the untransformed rice grains (Hagan et al., 2003). Methionine and cysteine levels were increased in transgenic rice by *E. coli* serine acetyltransferase isoform (*EcSAT*). Here cysteine, glutathione, free methionine and methionine content were elevated 2. 4-, 2-, 1. 4-, and 4.8-fold respectively. Moreover, the transgenic rice lines had ∼2 fold higher isoleucine, leucine and valine contents indicating potential alteration of methionine to isoleucine thus enhancing the nutritive value of rice (Nguyen et al., 2012).

## Manipulation of Other Protein Content

Glycosylated phaseolin, the main reserve globulin of common bean seeds is targeted to the vacuole and enclosed into the protein body (Bollini and Chrispeels, 1978). Since the overall lysine (3.4%) content in rice seeds makes it the first limiting amino acid, the bean β-phaseolin, with comparatively elevated lysine content (6.0%), was introduced into transgenic rice to increase the nutritional quality of grains (Zheng et al., 1995). The authors reported the biochemical, genetic and subcellular localization studies of the common bean phaseolin in the endosperms of transgenic rice which revealed the 4% phaseolin content in total rice endosperm protein with a positive gene dosage effect. Nutrition quality of rice is greatly affected by grain protein content (GPC). It was reported that qGPC-1 and qGPC-10, two stable quantitative trait loci are controlling GPC in a mapping population derived from the crossing of japonica and indica rice cultivars (Yang et al., 2019). Map-based cloning reveals that the gene encoding a glutelin type-A2 precursor is the key gene underlying qGPC-10 which functions as a positive regulator of grain protein content and is linked to the rice grain quality (Yang et al., 2019). Furthermore, it was evident that the single nucleotide protein present in the promoter of OSGluA2 is associated with grain protein content diversity (Yang et al., 2019).

A list of efforts attempted for enhancement of proteins and amino acid contents in rice has been presented in Table 4.

**TABLE 4:** Enhancement of proteins and amino acid contents in rice through transgenic method.

Proteins and amino acids			
Element type	Gene details	Sources	References
Tryptophan	Anthranilate synthase, OASA2	Rice	Wakasa et al., 2006; Saika et al., 2011
Lysine	Enhancement of lysine content through recombinant tRNA (lysine), Beta-Phaseolin with Lysine, aspartate kinase and dihydrodipicolinate synthase, lysine-rich binding protein accumulation, LRP, RLRH1, and RLRH2	Rice, Bacteria, Psophocarpus tetragonolobus	Zheng et al., 1995; Wu and Chen, 2003; Kawakatsu et al., 2010; Wong et al., 2015; Liu et al., 2016; Yang et al., 2016
Methionine and cysteine	Serine acetyltransferase, Sunflower seed albumin	*E. coli*, Sunflower	Hagan et al., 2003; Nguyen et al., 2012
Threonine, lysine, total amino acids, and crude protein	lysine (K)/threonine (T) motif (TKTKK1 and TKTKK2)	Rice	Jiang et al., 2016
Total protein and essential amino acids	Seed albumin (AmA1)	Amaranthus hypochondriacus	Xu et al., 2017
Phenylalanine and tryptophan	Phenylalanine biosynthetic enzyme	Rice	Yamada et al., 2008

## Manipulation of Starch Content

Starch-rich rice is a major diet of most of the world population. Over 2000 million populace in Asia intake more than 60% of their daily calories from rice (FAO, 2004). High amylose content in grains is a resource of resistant starch (Jiang et al., 2010). Studies have been shown that resistant starch has a protective effect against colorectal cancer in humans, pigs and rats (Phillips et al., 1995; Hasjim et al., 2010; Brites et al., 2011; Regmi et al., 2011). High amylose content also has a positive effect in some other health conditions like the development of non-reversible insulin resistance, depresses plasma total lipid, triacylglycerol and cholesterol concentrations (Lopez et al., 2001; Cheng and Lai, 2000; Fuentes-Zaragoza et al., 2011). Zhu et al. (2012) generated transgenic indica rice plants with high amylase (64.8%) content and high resistance through complete inhibition of the expression of two starch branching enzyme named SBEI and SBEIIb.

Transgenic grain feeding test on a rat showed a significant decrease in body weight gain, lowered fecal pH, increased fecal mass, increased fecal moisture and increased short-chain fatty acids. Zhu et al. (2012) also revealed that transgenic high-amylose rice possessed a positive effect in lowering the level of blood glucose in diabetic rats.

## Manipulation at Micronutrient Level

Micronutrient deficiency (MND) is a very common phenomenon of human health worldwide. The term “hidden hunger” is applied to designate the inadequacy of key micronutrients in the human daily diet. Inadequacy of micronutrient causes various diseases such as blindness, anemia, growth retardation, birth effects, and poor development of mental health (Howson et al., 1998; Oakley et al., 2004; Darnton-Hill et al., 2005). Morbidity and mortality through infection are also the effects of MND in various cases (Singhal and Austin, 2002; Black, 2003). Fe, Zn, Cu, and Mn are essential elements for the growth of living organisms as they are the important co-factors for many enzymes.

Rice is not considered as a high source of micronutrients in polished/edible form. Carbohydrates account for almost 90% of total dry matter in rice endosperm. Moreover, aleurone layer is oil-rich and hence removed or else it makes rice seed rancid upon storage. Essential micronutrients were removed during such processing of milling. Any enhancement in micronutrient content has a significant effect on human nutrition and health (Gregorio, 2002). A small amount of micronutrients (measured by plasma-optical emission spectrometry or ICP-OES) are present in the aleurone layer/bran of rice (Molina et al., 2019). As a consequence, over the last few years, several initiatives were taken to enhance some vital micronutrients such as vitamin A, iron, zinc, iodine and folate in rice through biofortification (Table 5; Impa et al., 2013; Swamy et al., 2016). Micronutrient enriched rice were developed either through traditional plant breeding or genetic engineering to combat the most vulnerable people such as resource-poor women, infants and children with deficiency diseases (Welch and Graham, 2004). Plants absorb minerals from the surrounding soil to meet their necessity rather than synthesizing them *de novo.* The Genetic engineering approach had been taken to enhance the essential micronutrients especially zinc and iron and vitamin A in rice. The transgenic approach increased nutrient content by enhancing the ability to uptake and translocation through the modulation of different ion transporters. Some anti-nutrient factors like phytic acids were reduced by gene insertion.

**TABLE 5:** Enhancement of micronutrients in rice through transgenic methods.

Micronutrients			
Element type	Gene details	Sources	References
Iron (Fe) and zinc (Zn)	Iron and zinc content elevation via ferritin, NAS, IDS3, OsVIT, IRT1, OsGluB1 pro-SoyferH1 gene	Soybean, Maize, Rice and Barley	Goto et al., 1999; Lucca et al., 2001; Vasconcelos et al., 2003; Qu et al., 2005; Suzuki et al., 2008; Masuda et al., 2009; Lee et al., 2009b, 2012; Paul et al., 2012; Oliva et al., 2013; Trijatmiko et al., 2016
Folate	Enhancement of folate content by AtGTPCHI, AtADCS, AtDHFS, AtFPGS, GTPCHI, ADCS, pterin and aminobenzoate	Arabidopsis	Storozhenko et al., 2007; Dong et al., 2014; Blancquaert et al., 2015
β-carotene	Phytoene synthase, crtI, locopene β cyclase, psy gene	Narcissus pseudonarcissus (daffodil),Erwinia uredovora,	Burkhardt et al., 1997; Ye et al., 2000; Tang et al., 2009
Iron (Fe), zinc (Zn) and beta carotene	AtNAS1, PvFERRITIN, CRTI, ZmPSY	Arabidopsis, Bean, Bacteria and Maize	Singh et al., 2017
Phytic acid	Lowering phytic acid content via lowering OsINO1 gene using Olesin18 promoter and Mutants with low PA (ipa)	Rice	Larson et al., 2000; Kuwano et al., 2009

## Manipulation of β-Carotene Content

Rice endosperm does not contain beta-carotene, thus vitamin A deficiency is common in the countries where rice is the staple food and meets the majority of the nutritional requirements of human health. Vitamin A or β-carotene is an essential micronutrient in a daily diet. Its’ deficiency cause a number of diseases such as night blindness, xerophthalmia and keratomalacia (Ye et al., 2000). Rice is devoid of provitamin A (β-carotene) or C_40_ carotenoid precursors in the endosperm. As an indispensable food crop it was essential to make rice more nutritious in terms of β-carotene. Burkhardt et al. (1997) designed an experiment which made rice endosperm capable of synthesizing β-carotene. After a thorough investigation using [^14^C] labeled substrate, it was found that rice endosperm has geranyl geranyl diphosphate (GGPP) (C_20_ general isoprenoid precursor necessary for C_40_ carotenoid biosynthesis). The first specific enzyme responsible for β-carotene synthesis in plants is phytoene synthase which condenses two molecules of GGPP. Taking the advantage of presence of GGPP in rice endosperm, a *japonica* variety of rice Taipei 309 was transformed by micro projectile bombardment with a cDNA coded for phytoene synthase from Daffodil or *Narcissus pseudonarcissus*. It was a significant achievement and first attempt to enable rice embryo for the synthesis of ∼2 μg of β-carotene per gram dry seed weight which is enough for young children. Ye et al. (2000) reported invention of Golden rice, which is known as prototype of GR1. They took psy (phytoene synthase) gene from daffodil and two other genes crtI (phytoene desaturase) and lcy (lycopene cyclase) from *E. uredovora*. Later studies (Schaub et al., 2005) showed that lcy is not required for beta-carotene biosynthesis in Golden rice. This is an important breakthrough where a biosynthetic pathway was established in rice endosperm and gave the rice grains a characteristic yellow color and named it “Golden Rice.” *Phytoene synthase* (*psy*) from daffodil, however, has a limitation for the synthesis of a higher level of carotenoids which was a limiting factor for desirable accumulation of β carotene. Subsequently, Paine et al. (2005) replaced the daffodil *psy* by its homolog from maize and thus improved the nutritional value of “Golden rice” by increasing the pro-vitamin A content by ∼23-fold higher which was as maximum as ∼37 μg/g in rice endosperm. They identified the limiting step of β carotene accumulation in some wild type tissues of different rice cultivars. A*psy* gene from maize was recognized which increased the carotenoid accumulation higher than previous Golden Rice and termed this as “Golden rice 2.” Golden rice is now considered as an effective source of vitamin A (Tang et al., 2009). After several years of its production Golden rice has become very popular and incorporated into many breeding programs in Asia (Potrykus, 2008). Compositional assessment of Golden Rice 2 event (GR2E) was achieved on the grain, straw and bran samples of GR2E and control rice (PSBRc82) collected from 4 locations in Phillipines. The level of β carotene was the only factor difference between two types of rice grains. GR2E can contribute up to 57–113% provitamin A for the preschool students in Bangladesh and Phillipines (Swamy et al., 2019).

Astaxanthin is a ketocarotenoid which is red in color with high antioxidant activity. Though, Most of the higher plants do not produce astaxanthin. Zhu et al. (2018) reported that bioengineering of astaxanthin biosynthetic pathway genes (sPaCrtI, sZmPSY1, sHpBHY, and sCrBKT, encode the enzymes phytoene desaturase, phytoene synthase, b-carotene hydroxylase, and b-carotene ketolase, respectively) could produce rice grains enriched with endospermic astaxanthin and had higher antioxidant activity. Tian et al. (2019) successfully enhanced biosynthesis of carotenoid in rice endosperm through metabolic engineering They showed that the expression of three chemically synthesized genes, i.e., tHMG1, ZmPSY1, and PaCRTI, considerably enhanced the amount of carotenoids in the endosperm of rice by improving the flux through the mevalonate route.

## Manipulation of Anthocyanin Content

Anthocyanins contain high antioxidant activities. It has been reported that engineering of eight anthocyanin related genes (two from maize which are regulatory genes and six from Coleus which are structural genes) with endosperm specific promoters produced a novel biofortified germplasm called “Purple Endosperm Rice” (Zhu et al., 2017). It has been proved that this novel rice endosperm has a high level of anthocyanin and antioxidant activity (Zhu et al., 2017).

## Manipulation of Folate Content

The deficiency of folate (vitamin B_9_) is a worldwide health problem as this water-soluble vitamin cannot be synthesized *de novo* by the human body. Only plants and microorganisms can synthesize folate. Hence humans have to depend exclusively on plant diet for folate supplements (Bekaert et al., 2008; Blancquaert et al., 2015). Inadequacy of folate can cause serious health issues such as neural tube defects (NTD), megaloblastic anemia, different ranges of cancer, some neurodegenerative disorder like Alzheimer’s disease, major depressive disorders (MDD) and a higher risk of cardiovascular and coronary diseases (Geisel, 2003; Li et al., 2003; Iyer and Tomar, 2009). The daily requirement of folate varies from 400 to 600 μg in men, women and pregnant women^1^. As an important staple food for all over the world rice lacks the required amount of folate to meet the daily diet. The concentration of folate was enhanced in rice by overexpressing two genes (pterin and aminobenzoate branches of folate biosynthetic pathway) encoding two enzymes from folate biosynthesis of *Arabidopsis thaliana* under control of a rice endosperm specific promoter (Storozhenko et al., 2007). These genetically modified rice lines were able to enhance folate level ∼100 times higher compared to the wild type plant thus recording about four-fold higher concentrations than the recommended daily intake (RDI) for folate^2^. In another study, the evaluation was done for each step of folate biosynthesis pathway in a *japonica* variety of rice (kitaake) by overexpression of two genes from *Arabidopsis thaliana* coding for GTP cyclohydrolase I and amino deoxychorismate synthase in the branches of pterin and *para*-aminobenzoate which affected the significant increase of folate content in rice endosperm (Dong et al., 2014).

## Manipulation of Iron and Zinc Content

Deficiency of iron is most common in the human population which leads to some important diseases such as anemia, impaired development and growth in preschool children and pregnant woman (World Health Organization [WHO], 2017). This crucial element plays a key role in electron transfer in both photosynthesis and respiration (Inoue et al., 2009; Ludwig and Slamet-Loedin, 2019). Biofortification of rice involves iron uptake, translocation and storage (Takagi et al., 1984). An iron storage protein “ferritin” is prevailing in most of the organisms including plants (Theil, 1987; Majumder et al., 2019). Soybean ferritin genes (*SoyferH1* and *SoyferH2*) were utilized for iron biofortification in rice as it was suggested that soybean ferritin iron complex is easily absorbed by the human intestine (Theil, 1987; Kok et al., 2018). The whole coding sequence of the soybean ferritin gene was introduced in rice (Kitake variety) by *Agrobacterium* mediated transformation (Goto et al., 1999). Rice endosperm specific promoters like globulin (*OsGlb*) and glutelin (*OsGluB1*) were used to enhance iron content in rice varieties which was able to increase nearly 3–3.7-fold iron content (Vasconcelos et al., 2003; Qu et al., 2005; Paul et al., 2012). Rice endosperm specific *GlutelinA*2 (*OsGluA2*) promoter was utilized to enhance Fe content in Pusa-sugandhi II (Paul et al., 2012). Overexpression of rice *ferritin* (*Osfer2*) increased 2.09-fold iron and 1.37-fold zinc in transgenic rice endosperm (Paul et al., 2012). IR68144 is a high iron rice variety (3.7-fold increased iron in polished grain) developed through conventional cross breeding between IR72 and Zawa Bonday (Gregorio et al., 2000). Vasconcelos et al. (2003) used IR68144 in transformation with ferritin genes from different sources as a recipient. IR68144 variety was also used for breeding with high yielding variety Swarna which increased 2.54-fold iron and 1.54-fold zinc than the wild type Swarna (Paul et al., 2012). Uptake of iron is based on two strategic types in plants, i.e., (1) reduction and (2) chelation (Ishimaru et al., 2006; Wirth et al., 2009; Kok et al., 2018). Under low iron conditions, graminaceous plants uptake iron by chelation based strategy where Fe^3+^ transports from rhizosphere to the roots of plants by producing phytosiderophores. However, rice uptake iron by the combined strategy of both chelation and the reduction. Under reduction based method Fe^3+^ is reduced to Fe^2+^ before going to absorb by plants. Usually, non-gramenaceous plants release protons to the surrounding environment which is the cause of low pH, help to the reduction of iron (Kok et al., 2018). During chelation based method nicotinamine synthase (NAS) is the principal enzyme for the release of phytosiderophores under iron deficit condition in rice plants. Transporter of mugeniec acid (MA) or TOM1 supports to form the phytosiderophores (Huguchi et al., 1999; Nozoye et al., 2011). But iron uptake in rice performed by the combined process of reduction and chelation (Kim and Guerinot, 2007). Since MA has a higher affinity toward Fe^3+^, it forms iron complexes and transported in the roots via *yellow stripe 1* transporters or *YSL1*. Hence keeping this view in mind scientists overexpressed genes with the involvement of MA for biofortification of iron content in rice such as overexpression of *NAS* genes. Overexpression of three *NAS* genes (*OsNAS1*, *OsNAS2*, and *OsNAS3*) increased iron content in rice (Table 5; Johnson et al., 2011; Lee et al., 2009a, 2012; Kok et al., 2018). Altered expression of *OsYSL2* increased 4.4-fold iron content in rice grains (Ishimaru et al., 2010). Moreover, in barley iron deficiency clone no.2 (IDS2) and no.3 (IDS3) performed an important role to tackle iron inadequacy. IDS genes activate mugineic acids which are highly expressed in roots during the iron deficit condition. IDS3 rice lines exhibited 1.4- and 1.3-fold elevated iron content in polished and brown rice grains respectively in both iron positive and negative soil (Masuda et al., 2008; Suzuki et al., 2008). Accumulation of zinc and iron content increased by knockdown of *OsVIT1* and *OsVIT2* (vacuolar iron transporter genes). This approach enhanced 1.4-fold iron in transgenic rice grains (Bashir et al., 2013). Another investigation made by Masuda et al. (2013b) where they introduced *SoyferH2* gene under the control of two promoters like *OsGluB1* and *OsGlb*and also with three barley genes (*HvNAS1, HvNAAT-A, HvNAAT-B*) encoding for the biosynthesis of mugineic acid in Tsukino Hikari rice cultivar. They found 4-fold increases in iron content in polished grains.

Zinc is another major micronutrient in rice act as a cofactor for ∼300 enzymes in the metabolism of lipids, proteins, carbohydrates and nucleic acids (Swamy et al., 2016). Malnutrition due to zinc is the major problem in the areas where polished rice is staple food (Rao et al., 2020). An appropriate amount of zinc is necessary for maintaining a healthy life. ∼7–13 mg zinc per day is needed for adults [Department of Health (United Kingdom) 1991; Institute of Medicine Food and Nutrition Board IMFNB 2001]. Zinc deficiency causes loss of appetite, retardation of growth, impaired immune function, loss of hair and weight, diarrhea, skin and eye lesions, delayed healing of wounds and mental lethargy (Prasad, 2004). For the last few years, efforts on zinc biofortification have been made to obtain high-zinc rice. Some candidate genes families were identified for their involvement in zinc and iron uptake such as *OsZIPs*, *OsIRTs*, *OsNASs*, and *OsNRAMPs*. Such investigations have been made to amplify zinc content in addition to iron by enhancing zinc uptake efficiency or unhindered transport in different plant tissues specifically during the grain filling stage in rice (Ishimaru et al., 2005, 2007, 2011; Chandel et al., 2010; Yamaji et al., 2013; Sasaki et al., 2014; Swamy et al., 2016). While reviewing the internal translocation of zinc in plant tissues it was observed by some scientists that variation of concentration of zinc uptake by root can cause a problem while passing zinc from vegetative tissues to grain (Stomph et al., 2014; Yin et al., 2016). Such studies based on zinc loading from vegetative tissues to rice endosperm remained very useful for the biofortification of zinc (Jiang et al., 2007; Wu et al., 2010). Several ZIP family metal transporter genes are upregulated under zinc deficient conditions in rice (Ramesh et al., 2003; Lee and An, 2009; Tan et al., 2015). *OsYSL2* gene overexpression caused 4-fold increases in iron and zinc content in rice (Ishimaru et al., 2010; Masuda et al., 2013a). Overexpression of *NAS* family genes enhanced iron and zinc concentration (Johnson et al., 2011; Lee et al., 2011). It was found that overexpression of OsIRT from rice and MxIRT from apple caused iron and zinc content enhancement in GM rice endosperm (Lee and An, 2009; Tan et al., 2015).

An initiative was taken by HarvestPlus in collaboration with International Rice Research Institute (IRRI) for better nutrition of crops to minimize micronutrient malnutrition by improving micronutrient content in staple crops. In 2013 they released a zinc biofortified rice variety in Bangladesh. The biofortified rice variety can meet 60% of daily zinc needs (Goldstein, 2018). Similarly in India the collaboration with the Department of Biotechnology (DBT), Indian Council of Agricultural Research (ICAR) and Indian Institute of Rice Research (IIRR) helped for the release of zinc biofortified rice varieties. Ninety-nine rice genotypes and 344 lines from different mapping populations have met the target (≥28 mg/kg of zinc). After the characterization of germplasm some zinc biofortified rice in polished grains has been released in India (Rao et al., 2020). DRR Dhan 45 is one of the released varieties with double the content of IR64 (22.6–24.0 ppm in polished grains) (accessed on 14 April 2019)^3^. Another drive made by Trijatmiko et al. (2016) where selected transgenic rice lines were field evaluated in two countries for biofortified iron and zinc content. Single locus insertion was observed for rice nicotinamine synthase (*OsNAS2*) and soybean ferritin (*SferH-1*) genes without any modified grain quality or any reduction in yield (Trijatmiko et al., 2016).

Besides iron content, Zn and Cd are also elevated in the mature seeds. As *OsIRT1* is an ion transporter involve in metal homeostasis (Lee and An, 2009). Three approaches (ferritin gene from *Phaseolus vulgaris*, phytase from *Aspergillus fumigatus* and cysteine peptides) were taken by Lucca et al. (2001) to increase iron content in transgenic rice and this attempt increased iron level about 130-fold. *Iron transporter I* and *Nicotinamine synthase* from *Arabidopsis and ferritin* gene from bean were introduced in rice to increase iron level in both polished and unpolished grains up to 10.46 μg/g dry weight.

## Biofortification of Multiple Micronutrients

A new strategy was adopted by Singh et al. (2017) to enhance iron, zinc and β-carotene in a single locus of rice endosperm. A transgenic rice line which expressed *Arabidopsis* nicotianamine synthase1 (At*NAS*1), bean *FERRITIN* (Pv*FERRITIN*), bacterial *CAROTENE DESATURASE* (*CRT*I), and maize *PHYTOENE SYNTHASE* (Zm*PSY*) showed enhanced iron, zinc and β-carotene level. A Significant amount of iron, zinc and β-carotene were increased in the endosperm. Zhu et al. (2019) discussed the latest advancement in the area of synthetic metabolic engineering of plants, exclusively the method of multigene stacking through considering genes from different/same metabolic pathways. Here, the aim was to enhance nutrient content and bioactive elements in the plants which will definitely meet the nutritional requirements of humans.

## Manipulation of Transporters

Rice has a distinctive root structure and mineral uptake system (Sasaki et al., 2016; Huang et al., 2020). The supply of mineral elements is mediated via transporters present in the nodal region of the plant (Yamaji and Ma, 2017). Several transporters involved in mineral nutrients uptake and their distribution have been identified in the last decade (Mitani-Ueno et al., 2018). Through the manipulation of Fe transporter, several efforts have been made to increase the concentration of Zn and Fe in rice grain (Masuda et al., 2012, 2009). It was found that introduction of rice Mn-NA transporter with barley NA^++^ synthase gene and soybean Ferritin gene resulted in 4.4-fold Fe increase and 1.6-fold Zn increase in transgenic rice grain (Masuda et al., 2012). Che et al. (2019) reported that OsVMT knockout resulted in increased Zn and Fe level in rice grain. Yamaji et al. (2017) reported that novel plasma membrane-localized transporter gene (OsSPDT) knockout resulted in ∼20% decrease of phytic acid in rice grains. Additionally, Knockout of OsSPDT also resulted in an increased level of Zn and Fe. Overexpression of rice peptide transporter NRT1 responsible for nitrate transport resulted in improved Se accumulation in rice grain (Zhang et al., 2019). Cd and As accumulation is toxic to the plant. Transgenic approaches have been adopted to develop knockout rice plants using OsNramp5, OsHMA2, and OsCd1 genes for reduction of the level of Cd and with the negative effect on grain yield (Ishikawa et al., 2012; Sasaki et al., 2012; Yamaji et al., 2013; Tang et al., 2017). On the other hand, Shao et al. (2018) showed that overexpreesion of OsHMA3 resulted in a significant reduction of Cd in rice without yield penalty. Overexpression of OsNIP1 and OsNIP3 in rice showed decreased As (arsenic) accumulation in the grain (Sun et al., 2018). Furthermore, As accumulation has been reduced by the targeted overexpression of OsABCC1 in the internode phloem and root cortical cells (Deng et al., 2018).

## Lowering Phytic Acid Content

*Myo* –inositol hexa phosphateor phytic acid (PA) is the principal storage form of phosphorus (P) in the cereal grains. PA is considered as the most abundant inositol phosphate and chelates metal ions such as calcium, magnesium, potassium, zinc and iron to form phytate or phytin. Cereals are very rich in phytic acid content. Rice contains 8.7% PA in their bran (Lehrfeld, 1994; Gupta et al., 2015). This insoluble salt prevents the absorption of important nutrients in the intestine of human beings which leads to the deficiency of micronutrients (Raboy, 2000; Mitchikpe et al., 2008; Perera et al., 2018). Hence, different methods of biofortification had been applied to lower down the phytic acid levels in rice to increase the absorption rate of iron and zinc (Hurrell et al., 2003). Mutants with low PA content (*lpa*) were characterized and developed which showed higher micronutrient content in the seed. 45% reduction of PA was seen due to a non-lethal mutant in Kaybonnet rice variety (Larson et al., 2000). Initiatives had been taken to lower down the level of *OsINO1* gene expression which automatically reduced PA using *Olesin18* (*Ole18*) promoters (Kuwano et al., 2009). As phytate is not digested by animal system due to lack of phytase enzyme, low phytate rice was generated very recently by overexpressing *E. coli* appA gene which enhanced inorganic P level by 4-fold along with zinc and iron. The gene *appA* encoded AppA protein which is non-glycosylated and periplasmic (Bhattacharya et al., 2019).

## Conventional and Molecular Breeding Strategies for the Improvement of Nutrients

To create superior cultivars of rice with higher nutritional value, scientists of IRRI and the University of Adelaide collaborated for documenting the zinc and iron content of several rice varieties (Graham et al., 1999). This work resulted in the identification of rice varieties with high iron and zinc content. The initial focus is on successfully breeding a rice variety containing higher absolute mineral content. Graham et al. (2007) tried to gain higher bio-availability by three possible ways (a) raise the percentage of bio-availability (by declining material that inhibits uptake of nutrient), (b) raise the nutrient concentration in the grain, and (c) a mixture of these two methods.

The breeding strategy involves screening of rice lines contain high amount of micronutrients necessary for the human population. Numerous works have been done based on the finding of rice germplasm with high amount of micronutrients (Anandan et al., 2011; Anuradha et al., 2012; Babu et al., 2012). It seems that major mineral content such as Fe, Zn, Cu, Mn was higher in traditional rice varieties in comparison to modern high yielding varieties (Anandan et al., 2011). Highest grain iron and zinc concentration were identified in some aromatic rice varieties (Jalmagna, Zuchem, and XuaBueNuo). Range of iron and zinc concentration in those aromatic rice cultivars were ∼18–22 μg/g and ∼24–35 μg/g respectively (Graham et al., 1999). Agronomic traits are studied and manipulated by QTL mapping for several years (Jeong et al., 2020). Genes responsible for micronutrients especially iron, zinc, and other essential micronutrient content in rice were mapped through QTL which will help in future breeding program. QTLs used in breeding programs to increase nutrient contents are tabulated in Table 6. Five QTL had been mapped in different chromosomes for iron and zinc concentration in unpolished/brown rice grains (Anuradha et al., 2012). Genome wide mapping was done from the cross of Madhukar × Swarna variety that helps in mapping 14 QTLs for iron and zinc content in unpolished rice grains (Agarwal et al., 2014). SNP and SSR markers were used in MAS to enhance zinc and iron content. Recently double-haploid (DH) mapping populations of rice were evaluated for higher grain zinc in rice (Swamy et al., 2018a). Swamy et al. (2018a) mapped QTLs for grain micronutrients in addition to yield potential and QTL x QTL interconnections. Some candidate genes associated with rice grain zinc (*OsNRAMP*, *OsNAS*, *OsZIP*, *OsYSL*, *OsFER*, and *OsZIFL* family) were identified in association with these QTLs. QTLs for other mineral elements were also mapped (Swamy et al., 2018a). Another rice DH population was used (Korean *japonica* cultivars) to identify the major effect of QTLs related to grain iron and zinc content (Jeong et al., 2020), where the candidate genes were also analyzed. Two double haploid rice lines were used for QTL analysis for 13 grain elements (Descalsota-Empleo et al., 2019). Lee et al. (2020) analyzed and produced a double haploid by crossing between an *indica* (93-11) and *japonica* (Milyang 352) rice cultivars. They mapped two co-localized QTL such as *qFe3-1 and qZn3-1* for iron and zinc respectively which will be useful for future nutrient-rich rice (Lee et al., 2020). Double haploid rice lines were investigated by Calayugan et al. (2020) by crossing IR05F102 x IR69428 for high grain Zn and Fe. Here, they found 5 QTL related to these micronutrients. A further study was made by Swamy et al. (2018b) which showed two BC_2_F_3_ mapping populations, the crosses between *O. sativa* (Swarna variety) and *O. nivara*, QTLs were mapped for Zn and Fe concentration in grains. A large population with 485 germplasm lines was assessed for the association mapping of Zn and Fe content in rice grains. In addition to this, iron and zinc controlling linkage disequilibrium QTL mapping was performed which were very useful for micronutrient rich milled rice (Pradhan et al., 2020). Cereal grains are one of the major sources of protein for human food. Hence, quantification of grain protein content (GPC) is essential (Peng et al., 2014). Prolamins and glutelins are the major sub-divisions of protein content in rice (Shewry et al., 1995). For human diet, glutelin have a high nutritive value (Ufaz and Galili, 2008; Zhang et al., 2008). A large number of QTLs associated with GPC has been identified and mapped in milled rice. All amino acids content (AAC) including essential and total AAC were characterized using a population of 190 recombinant inbred lines (RILs) from a cross between Zhenshan97 and Nanyangzhan rice variety (Wang et al., 2008). It was proved that a QTL for AAC, *qPC1* encodes a putative amino acid transporter *OsAAP6* (Peng et al., 2014). Very recently, another QTL for GPC in rice was mapped in more than one chromosome like 1, 2, 6, 7, 10, and 11. Three QTLs were identified of which one is responsible for grain protein content (*qGPC1.1*) and the other two for single grain protein content (*qSGPC2.1 and qSGPC7.1*). Moreover, these QTLs were detected as environmentally stable. A glutelin encoded gene (*Os01g0111900*) was found to be associated with this QTL, was upregulated during seed development (Chattopadhaya et al., 2019).

**TABLE 6:** QTLs identified for nutrient enhancement in rice (Oryza sativa).

Traits	Respective QTL	References
Zinc (Zn)	qZN-5, qZN-7, qSZn2, qSZn12, qZn7, qZn3.1, qZn7.1, qZn7.2, qZn7.3, qZn12.1, qZn4, qZn6, qZn1-1, qZn12-1, qZn5-1, qZn8-1, qZn12-1, qZn2.1, qZn2.1, qZn3.1, qZn6.1, qZn6.2, qZn8.1, qZn11.1, qZn12.1, qZn12.2, qZn2.2, qZn8.3, qZn12.3, qZn3.1, qZn7, qZn8.3, qZn3-1, qZn1.1, qZn5.1, qZn9.1, qZn12.1, qZn1.1, qZn6.1, qZn6.2, qZn2-1, qZn2-2, qZn5, qZn10, qZn2.1, qZn3.1, qZn5.1, qZn5.2, qZn7.1, qZn9.1, qZn11.1, qZn 1.1, qZn2.1, qZn3.1, qZn3.2, qZn5.1, qZn6.1, qZn8.1, qZn8.2, qZn9.1, qZn12.1	Lu et al., 2008; Garcia-Oliveira et al., 2009; Ishikawa et al., 2010; Zhang et al., 2011; Anuradha et al., 2012; Jin et al., 2013; Swamy et al., 2018a; Swamy et al., 2018b; Dixit et al., 2019; Descalsota-Empleo et al., 2019; Calayugan et al., 2020; Lee et al., 2020; Pradhan et al., 2020
Iron (Fe)	*qFE-1, qFE-9, qGFe4, qSFe1, qSFe12, qFe1, qFe3, qFe6, qFe2-1, qFe9-1, qFe4.1, qFe3.3, qFe7.3, Fe8.1, qFe12.2, qFe3-1, qFe9.1, qFe12.1, qFe1.1, qFe1.2, qFe6.1, qFe6.2, qFe5, qK6.1, qFe2.2, qFe3.1, qFe4.1., qFe6.1, qFe8.1, qFe11.2, qFe11.3, qFe12.1*,	Stangoulis et al., 2007; Lu et al., 2008; Garcia-Oliveira et al., 2009; Ishikawa et al., 2010; Norton et al., 2010; Jin et al., 2013; Swamy et al., 2018a; Swamy et al., 2018b; Dixit et al., 2019; Calayugan et al., 2020; Lee et al., 2020; Pradhan et al., 2020
Zn and Iron	*qZn10, qZn2, qZn4, qZn5, qZn6, qZn7, qZn9*	Zhang et al., 2014
Manganese (Mn)	*qMn1-1, qMn2-1, qMn3-1, qMn10-1, qMn2.1, qMn2.1, qMn7.1, qMn1.1, qMn1.2, qMn3.1, qMn3.2, qMn4.1*	Garcia-Oliveira et al., 2009; Swamy et al., 2018a; Descalsota-Empleo et al., 2019
Calcium	*qCa1-1, qCa4-1, qCa5-1, qCa9-1, qCa10-1, qCa11-1, qCa12-1, qCa1.1, qCa1.1, qCa2.1, qCa2.1, qCa3.1, qCa3.2*,	Garcia-Oliveira et al., 2009; Swamy et al., 2018a
Magnesium (Mg)	*qMg1-1, qMg3-1, qMg5-1, qMg9-1, qMg12-1, qMg3.1, qMg3.2, qMg5.1, qMg8.1, qMg9.1, qMg1.1, qMg7.1, qMg8.1, qMg11.1*	Garcia-Oliveira et al., 2009; Swamy et al., 2018a; Descalsota-Empleo et al., 2019
Phosphorus (P)	*qP1-1, qP3-1, qP8-1, qP9-1, qP12-1, qP1.1, qP2.1, qP2.2, qP5.1, qP6.1, qP11.1, qP11.2*	Garcia-Oliveira et al., 2009; Swamy et al., 2018a; Descalsota-Empleo et al., 2019
Potassium (K)	*qK1-1, qK4-1, qK8-1, qK9-1, qK2.1, qK4.1, qK4.2, qK5.1, qK9.1, qK3.1, qK3.2, qK3.3, qK4.1, qK5.1*,	Garcia-Oliveira et al., 2009; Swamy et al., 2018a; Descalsota-Empleo et al., 2019
Boron (B)	*qB2.1, qB3.1, qB4.1, qB4.2, qB10.1*	Swamy et al., 2018a; Descalsota-Empleo et al., 2019
Cobalt (Co)	*qCo1.1, qCo3.1, qCo4.1, qCo12.1, qCo7.1, qCo10.1*	Swamy et al., 2018a; Descalsota-Empleo et al., 2019
Copper (Cu)	*qCu3.1, qCu4.1, qCu4.2, qCu1.1, qCu1.2, qCu2.1, qCu6.1, qCu8.1*	Swamy et al., 2018a; Descalsota-Empleo et al., 2019
Molybdenum (Mo)	*qMo1.1, qMo1.1, qMo1.2, qMo1.3, qMo1.3, qMo2.1, qMo11.1, qMo12.1, qMo12.1, qMo12.1, qMo1.1*	Swamy et al., 2018a
Sodium (Na)	*qNa1.1, qNa1.2, qNa7.1, qNa7.2, qNa10.1, qNa3.1, qNa11.1, qNa11.2*	Swamy et al., 2018a; Descalsota-Empleo et al., 2019
Phytate	*QTL for phytate*	Stangoulis et al., 2007
Grain protein content (GPC)	*QTL for PC (Protein content). qPC1, qPC2, qPC3, qPC6.1, qPC6.2, qPC8, qPC12.1, qPC1.1, qPC11.1, and qPC11.2, qPC-3, qPC-4, qPC-5, qPC-6 and qPC-10, qPr1 and qPr7, qPro-8, qPro-9 and qPro-10, qGPC1.1, qSGPC2.1 and qSGPC7.1*	Tan et al., 2001; Qin et al., 2009; Yu et al., 2009; Zhong et al., 2011; Yun et al., 2014; Mahender et al., 2016; Kinoshita et al., 2017; Chattopadhaya et al., 2019
Amino acid content (AAC)	*qAa1, qAa7*	Zhong et al., 2011
Lysine	*qAa9, qPC1*	Zhong et al., 2011; Peng et al., 2014
Cys/Leu/Ile/Phe	*qAA.10*	Wang et al., 2008

## Agronomic Biofortification

Apart from transgenic methods, molecular and conventional breeding approaches, agronomic biofortification is another strategy based on application of optimized fertilizer for nutrient enhancement in rice. Microelements like zinc, iron, copper manganese etc. are generally absorbed from the soil in different plants (Garg et al., 2018). The application of fertilizers in the soil can also meet the deficiency of micronutrients in plants (Graham et al., 2007). Zinc was applied through soil and foliar methods which increased maximum yield with higher Zn content in rice grains. Furthermore, high Zn accumulation resulted in higher iron accumulation and loss of phytic acid (Saha et al., 2017). Application of nitrogen fertilizer enhanced GPC, Zn and Fe in brown rice. Different locations altered the elevation of micro and macronutrients in rice by the application of N_2_ fertilizer (Chandel et al., 2010).

## Conclusion and Future Prospective

Rice is a key source of carbohydrate and B vitamins. However, rice consumption as a major food is not sufficient to meet the nutrition requirement for rice eaters in developing countries and most of the people of rice dependent countries experience various forms of undernourished feeding. Agriculture pattern, post-harvest processes and climate conditions have a considerable negative effect on the nutritional quality of rice. Enhancement of rice nutritional value is necessary in developing countries to avoid malnutrition in the coming era. In current era, rice researchers along with rice nutritionists are working together toward optimizing the nutrition level of rice by adopting biotechnological or breeding methods to get new and better varieties and to provide the best to the rice dependent population. Recent progress in rice nutrition enhancement through biotechnology might be capable to ameliorate malnutrition presently experienced. Nutritional value related genes and QTLs will play crucial roles in developing the required genotypes. In current years, considerable efforts have been made in molecular studies on grain amino acid and protein content, glycemic index value, vitamins, minerals and their transporters, phytic acid, phenolic and flavonoid compounds, zinc and iron content. However, more research is needed for the processing of the newly developed nutritionally enhanced varieties. In India, recently released zinc rich and high protein rice varieties gives the optimistic message on the positive and forward move in rice crop enhancement program. The transgenic method will additionally support to improve grain nutrition to the desired level satisfactorily. On the basis of present evolutionary idea, it is believed that some transgenes from rice are not unsafe for the environment. On the other hand, some transgenes which do not have major selective advantages could cause partial or potential environmental problems. However, it is important to raise awareness of the factors influencing nutrient composition in the newly developed varieties. These newly developed rice varieties should undergo whole nutrient testing, environmental benefits and risks such as the impact on human health, environmental assessments and public accessibility in order to mark the standard for considering the impact of new varieties.

## Author Contributions

PD and AL generated the concept and made the outline of the review. PD and SA wrote the draft manuscript and prepared the tables and figures. AL finalized the manuscript. All authors contributed to the article and approved the submitted version.

## Conflict of Interest

The authors declare that the research was conducted in the absence of any commercial or financial relationships that could be construed as a potential conflict of interest.

## Footnotes

1 http://ods.od.nih.gov/Health_Information/Dietary_Reference_Intakes.aspx

2 http://ods.od.nih.gov/factsheets/Folate-Health Professional/

3 https://icar.org.in/node/6293
